# New Bounds on Capacity Region of Multiple Access Channels in Visible Light Communications

**DOI:** 10.3390/e24091233

**Published:** 2022-09-02

**Authors:** Ruixin Yang, Bing Li, Shuai Ma, Youlong Wu, Zongyan Li, Shiyin Li

**Affiliations:** 1 School of Information and Control Engineering, China University of Mining and Technology, Xuzhou 221116, China; 2 Engineering Research Center of Intelligent Control for Underground Space, Ministry of Education, China University of Mining and Technology, Xuzhou 221116, China; 3 National Mobile Communications Research Laboratory, Southeast University, Nanjing 210096, China; 4 Shaanxi Key Laboratory of Information Communication Network and Security, Xi’an University of Posts & Telecommunications, Xi’an 710121, China; 5 School of Information Science and Technology, ShanghaiTech University, Shanghai 201210, China

**Keywords:** visible light communications, multiple access channel, channel capacity region

## Abstract

In this paper, we propose new inner and outer bounds of the capacity region for multiple access channels in visible light communication (VLC) networks under both peak and average optical power constraints. Specifically, the proposed inner bounds are established by employing the single-user capacity achieving input distribution for each user. The proposed outer bounds are derived by determining single-user capacities for each user and calculating a sum capacity upper bound by relaxing the input constraints. Numerical results show that the proposed new bounds are extremely tight and outperform existing bounds over wide ranges of SNRs.

## 1. Introduction

Enjoying a large range of license-free bandwidth, visible light communication (VLC) has received increasing interests [1,2,3] as a complementary technology to conventional radio frequency (RF) communication, which is suffering from a spectrum crisis. By utilizing existing light-emitting diodes (LEDs) as transmitters, the VLC system can simultaneously provide both illumination and communication but also has other several advantages, such as ultra low electromagnetic radiation, strong transmission security, and high energy efficiency. As a wireless broadband technology, it is essential for the VLC network to support multiple users simultaneously. Such a scenario can be mathematically described by the multiple access channel model in information theory, where multiple transmitters transmit individual information to one common receiver at the same time. The capacity region of multiple access channels can characterize fundamental limits of achievable rates, and thus, it serves as the theoretical basis for the other practical VLC network designs.

Note that VLC exploits the intensity modulation and direct detection (IM/DD) scheme, and the information is represented by intensity of signals. Thus, mathematically, the transmitted signals of VLC must be real and non-negative, which is different from the RF complex-valued signals. Moreover, due to human eye safety and illumination considerations, both the peak and average optical power of transmitted signals are limited. Therefore, such a situation makes the classic Shannon capacity formula with Gaussian input [4] not applicable to VLC networks. A great many efforts have been made on studying the point-to-point channel [5,6] or broadcast channel (BC) [7,8], while some works [9,10] have begun to study the multiple access channels. In [9], both the inner and outer bounds have been established for VLC multiple access channel networks. Specifically, the outer bounds were derived based on the results for single-user optical intensity channels; the inner bounds were obtained by assuming that the input follows the truncated Gaussian and uniformly-spaced discrete distributions, respectively. The bounds provide a capacity approximation within a constant gap at a high signal-to-noise ratio (SNR) and characterize the capacity region at low SNR. Under a per-user average or peak-power constraint, both inner and outer bounds on the capacity region of the optical intensity multiple access channels are presented in [10], where the bounds are asymptotically tight at high SNR. To the best of the authors’ knowledge, the exact capacity region of VLC multiple access channels and the optimal input distribution remains unknown so far.

In this work, we investigate the capacity region of the VLC multiple access channels with both peak and average power constraints. Due to the peak optical power constraint, the optimal input follows discrete distributions. By assuming the discrete input, finding the exact channel capacity region of VLC multiple access channels is formulated as a mixed discrete optimization problem, which is non-convex due to the objective function without analytical expression. We adopt the inexact gradient method to overcome this challenge. Furthermore, by restricting and relaxing the discrete inputs, we develop both inner bounds and outer bounds of the channel capacity of VLC multiple access channels, respectively, which are shown to be tighter than the existing benchmarks.

The rest of this paper is as follows: Section 2 presents the two-user system model of the typical optical intensity multiple access channels under both the peak and average power constraints and the details of the proposed inner and outer bounds. Section 2.1 gives the derivation of the exact single-user channel capacity $I\left(X_{i};Y|X_{\bar{i}}\right)$. Section 2.2 shows the inner bound of sum capacity $I\left(X_{1},X_{2};Y\right)$; Section 2.3 shows the outer bound of sum capacity $I\left(X_{1},X_{2};Y\right)$. Then, the numerical simulation results are provided in Section 3 to demonstrate the effectiveness of our approach. Finally, Section 4 draws the conclusions of this study.

Notations: $I\left(X;Y\right)$ denotes the mutual information between random variables *X* and *Y*. $H\left(X\right)$ denotes the entropy of random variable *X*. $h\left(X\right)$ denotes the differential entropy of random variable *X*.

## 2. Capacity Region of VLC Multiple Access Channel Networks

**Figure 1:**
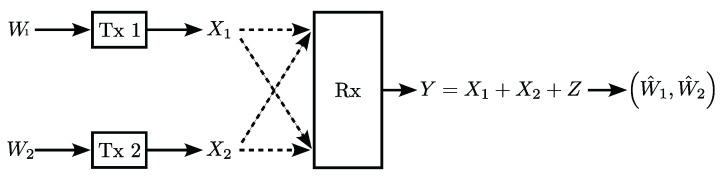
Optical multiple-access channel with two transmitters.

Consider a typical optical intensity multiple access channels, as shown in Figure 1, where the system contains two transmitters (Tx 1 and Tx 2) and one receiver (Rx). Each transmitter is installed with a single LED, and the receiver is equipped with a single photon detector (PD). $W_{1}$ and $W_{2}$ are the input information of Tx 1 and Tx 2. Let $X_{1}$ and $X_{2}$ denote the transmitted signals of transmitter 1 and transmitter 2, respectively. Since the information is embedded in the intensity of the optical signal, $X_{1}$ and $X_{2}$ should be real and non-negative. Additionally, due to eye safety standards and practical illumination requirements, both the peak and average optical power should be restrained, such that $0\leq X_{1}\leq A_{1}$, $E\left\{X_{1}\right\}\leq \mu_{1}$, $0\leq X_{2}\leq A_{2}$, and $E\left\{X_{2}\right\}\leq \mu_{2}$. We consider a deterministic VLC channel model, where the channel gain between the two transmitters and the receiver is fixed as 1. Over the multiple access channels [4], the received signal *Y* is given by

(1) $$\begin{array}{c} Y=X_{1}+X_{2}+Z, \end{array}$$

where *Z* is the independent Gaussian noise with mean zero and variance $\sigma^{2}$, and the Rx can output the decoding results $\left({\hat{W}}_{1},{\hat{W}}_{2}\right)$.

To characterize the capacity region of VLC multiple acces channels, let $P_{X_{i}}\left(x_{i}\right)$ denote the distribution of $X_{i}$, and $R_{i}$ denote the achievable rate from transmitter *i*, where i=1,2. Then, the capacity region of the VLC multiple access channels is the convex closure of $\underset{P_{X_{1}}\left(x_{1}\right),P_{X_{2}}\left(x_{2}\right)}{⋃}R\left(X_{1},X_{2}\right)$, where $R\left(X_{1},X_{2}\right)$ is the set of rate pairs $\left(R_{1},R_{2}\right)$, such that [4]

(2) $$\begin{array}{c} \left\{\begin{array}{c} R_{1}\leq I\left(X_{1};Y|X_{2}\right)\\ R_{2}\leq I\left(X_{2};Y|X_{1}\right)\\ R_{1}+R_{2}\leq I\left(X_{1},X_{2};Y\right) \end{array}\right. \end{array}$$

for a fixed product distribution $P_{X_{1}}\left(x_{1}\right)P_{X_{2}}\left(x_{2}\right)$ satisfying input constraints.

Due to the limited amplitude (peak optical power constraint), the optimal input distributions of (2) should be discrete over a finite set of points [11]. Unfortunately, there is no efficient method other than exhaustive search [11] to find channel capacity of (2). In the follows, we will present an efficient method to find capacity-achieving discrete input distributions $P_{X_{i}}^{*}\left(x_{i}\right)$, which can maximize the mutual information $I\left(X_{i};Y|X_{\bar{i}}\right),i=1,2,$$\bar{i}=3-i$. Based on this method, we also can obtain a new inner bound and a new outer bound for $I\left(X_{1},X_{2};Y\right)$.

### 2.1. Exact Single-User Channel Capacity $I\left(X_{i};Y|X_{\bar{i}}\right)$

To find the optimal distribution, we assume that the signal $X_{i}$ follows discrete distribution with $K_{i}$ non-negative real values ${\left\{x_{i,j}\right\}}_{1\leq j\leq K_{i}}$, where $K_{i}\geq 2$ denotes the number of mass points. The discrete input distribution should satisfy some constraints, as follows:

(3a) $$\begin{array}{cc} & \mathrm{Pr}\left\{X_{i}=x_{i,j}\right\}=p_{i,j}\geq 0,0\leq x_{i,j}\leq A_{i},\forall j\in K_{i}, \end{array}$$

(3b) $$\begin{array}{cc} & E\left\{X_{i}\right\}=\sum_{j=1}^{K_{i}}p_{i,j}x_{i,j}\leq \mu_{i}, \end{array}$$

(3c) $$\begin{array}{cc} & \sum_{j=1}^{K_{i}}p_{i,j}=1, \end{array}$$

where $x_{i,j}$ is the *j*th point of $X_{i}$, and $p_{i,j}$ is the corresponding probability, $K_{1}≜\left\{1,2,...,K_{1}\right\}$ and $K_{2}≜\left\{1,2,...,K_{2}\right\}$.

Then, we have

(4a) $$\begin{array}{cc} C_{i} & \overset{\Delta }{=}\max_{P_{X_{i}}\left(x_{i}\right)}I\left(X_{i};Y|X_{\bar{i}}\right) \end{array}$$

(4b) $$\begin{array}{cc} & =\max_{P_{X_{i}}\left(x_{i}\right)}h\left(Y_{i}\right)-h\left(Z\right) \end{array}$$

(4c) $$\begin{array}{cc} & =\max_{P_{X_{i}}\left(x_{i}\right)}-\int_{-\infty }^{\infty }f_{Y_{i}}\left(y_{i}\right)\log_{2}f_{Y_{i}}\left(y_{i}\right)dy_{i}-\frac{1}{2}\log_{2}2\pi e\sigma^{2} \end{array}$$

where $Y_{i}\overset{\Delta }{=}X_{i}+Z$ for i=1,2.

Since the noise *Z* follows the Gaussian distribution, the probability density function (PDF) $f_{Y_{i}}\left(y_{i}\right)$ can be written as

(5) $$\begin{array}{c} f_{Y_{i}}\left(y_{i}\right)=\frac{1}{\sqrt{2\pi }\sigma }\sum_{j=1}^{K_{i}}p_{i,j}e^{-\frac{{\left(y_{i}-x_{i,j}\right)}^{2}}{2\sigma^{2}}}. \end{array}$$

Therefore, finding the capacity of the VLC multiple access channels (4c) can be mathematically formulated as the following optimization problem:

(6) $$\begin{array}{cc} \min_{K_{i},\left\{p_{{}_{i,j}}\right\},\left\{x_{i,j}\right\}} & \int_{-\infty }^{\infty }f_{Y_{i}}\left(y_{i}\right)\log_{2}f_{Y_{i}}\left(y_{i}\right)dy_{i}\\ s.t. & (3a),(3b),(3c). \end{array}$$

Problem (6) is a mixed discrete and nonconvex problem, which is convex with respect to the continuous variable $\left\{p_{i,j}\right\}$ but is non-convex over discrete variables $K_{i}$ and $\left\{x_{i,j}\right\}$. Moreover, there is no analytic expression of the objective function (6). Hence, problem (6) is difficult to solve. To overcome this challenge, the inexact gradient descent method [12] is applied to problem (6), and we obtain both the optimal input distribution $P_{X_{i}}^{*}\left(x_{i}\right)$, i.e., $\left\{K_{i}^{*},\left\{p_{i,j}^{*}\right\},\left\{x_{i,j}^{*}\right\}\right\}$, and the channel capacity $C_{i}$.

### 2.2. Inner Bound of Sum Capacity $I\left(X_{1},X_{2};Y\right)$

We define $C_{1,2}\overset{\Delta }{=}\max_{P_{X_{1}}\left(x_{1}\right),P_{X_{2}}\left(x_{2}\right)}I\left(X_{1},X_{2};Y\right)$. Thus, we have

(7) $$\begin{array}{cc} C_{1,2} & =\max_{P_{X_{1}}\left(x_{1}\right),P_{X_{2}}\left(x_{2}\right)}h\left(Y\right)-h\left(Z\right), \end{array}$$

where the PDF $f_{Y}\left(y\right)$ is given as

(8) $$\begin{array}{c} f_{Y}\left(y\right)=\frac{1}{\sqrt{2\pi }\sigma }\sum_{m=1}^{K_{1}}\sum_{n=1}^{K_{2}}p_{1,m}p_{2,n}e^{-\frac{{\left(y-x_{1,m}-x_{2,n}\right)}^{2}}{2\sigma^{2}}}. \end{array}$$

Different from problem (6), the capacity region of the VLC multiple access channels (7) involves two variables, $P_{X_{1}}\left(x_{1}\right)$ and $P_{X_{2}}\left(x_{2}\right)$, which makes the optimal solutions hard to find. Thus, we have to find the suboptimal solutions. Specifically, we substitute the distributions $P_{X_{1}}^{*}\left(x_{1}\right)$ and $P_{X_{2}}^{*}\left(x_{2}\right)$ of problem (6) into the right-hand side of (7), and the obtained result can be served as an inner bound of $C_{1,2}$.

### 2.3. Outer Bound of Sum Capacity $I\left(X_{1},X_{2};Y\right)$

Let us define $\hat{X}\overset{\Delta }{=}X_{1}+X_{2}$. Then, we have $0\leq \hat{X}\leq \hat{A}$ and $E\left\{\hat{X}\right\}=E\left\{X_{1}\right\}+E\left\{X_{2}\right\}\leq \hat{\mu }$, where $\hat{A}\overset{\Delta }{=}A_{1}+A_{2}$ and $\hat{\mu }\overset{\Delta }{=}\mu_{1}+\mu_{2}$. Assume that the signal $\hat{X}$ follows a discrete distribution with $\hat{K}$ non-negative real values ${\left\{{\hat{x}}_{k}\right\}}_{1\leq k\leq \hat{K}}$, where $\hat{K}\geq 2$ denotes the number of mass points, such that

(9a) $$\begin{array}{cc} & \mathrm{Pr}\left\{\hat{X}={\hat{x}}_{k}\right\}=p_{k}\geq 0,0\leq {\hat{x}}_{k}\leq \hat{A},\forall k\in \hat{K}, \end{array}$$

(9b) $$\begin{array}{cc} & E\left\{\hat{X}\right\}=\sum_{k=1}^{\hat{K}}p_{k}{\hat{x}}_{k}\leq \hat{\mu }, \end{array}$$

(9c) $$\begin{array}{cc} & \sum_{k=1}^{\hat{K}}p_{k}=1, \end{array}$$

where $\hat{K}\overset{\Delta }{=}\left\{1,2,...,\hat{K}\right\}$. Thus, the outer bound of $C_{1,2}$ is given by

$$\begin{array}{cc} & C_{1,2}\leq \max_{P_{\hat{X}}\left(\hat{x}\right)}-\int_{-\infty }^{\infty }f_{Y}\left(y\right)\log_{2}f_{Y}\left(y\right)dy-\frac{1}{2}\log_{2}2\pi e\sigma^{2}. \end{array}$$

By relaxing the input constraints (3) to (9), the outer bound of $C_{1,2}$ can be formulated as

(10) $$\begin{array}{cc} \min_{\hat{K},\left\{p_{k}\right\},\left\{{\hat{x}}_{k}\right\}} & \int_{-\infty }^{\infty }f_{Y}\left(y\right)\log_{2}f_{Y}\left(y\right)dy\\ s.t. & \;(9a),(9b),(9c), \end{array}$$

which is a mixed discrete and nonconvex problem.

Likewise, the inexact gradient descent method [12] can be used to handle problem (10) and obtain the outer bound of channel capacity $C_{1,2}$. Note that the above capacity region of the two-users case can be directly extended to the *N*-user (N≥3) multiple access channel.

## 3. Results and Discussion

To evaluate the obtained capacity region in the previous section, the outer bound ${\bar{C}}^{\left[1\right]}$ based on Equation (12) if $\alpha_{i}\in \left(0,\frac{1}{2}\right)$ and Equation (21) if $\alpha_{i}\in \left(\frac{1}{2},1\right)$ in [5], outer bound ${\bar{C}}^{\left[2\right]}$ based on theorem 1 in [6], outer bound ${\bar{C}}^{\left[3\right]}$ based on Equation (13) if $\alpha_{i}\in \left(0,\frac{1}{2}\right)$ and Equation (22) if $\alpha_{i}\in \left(\frac{1}{2},1\right)$ in [5], outer bound ${\bar{C}}^{\left[4\right]}$ from proposition 5 in [10], inner bound ${\bar{C}}^{\left[5\right]}$ from proposition 6 in [10], and the discrete entropy maximization inner bound in [13] are presented for comparison, where $\alpha_{i}\overset{\Delta }{=}\frac{\mu_{i}}{A_{i}}$ for all *i*. Note that both the outer bound ${\bar{C}}^{\left[4\right]}$ and inner bound ${\bar{C}}^{\left[5\right]}$ only fit for specific case, i.e., with only the peak optical power constraint, and the discrete entropy maximization inner bound in [13] is first extended to the VLC multiple access channel scenario in this paper.

**Figure 2:**
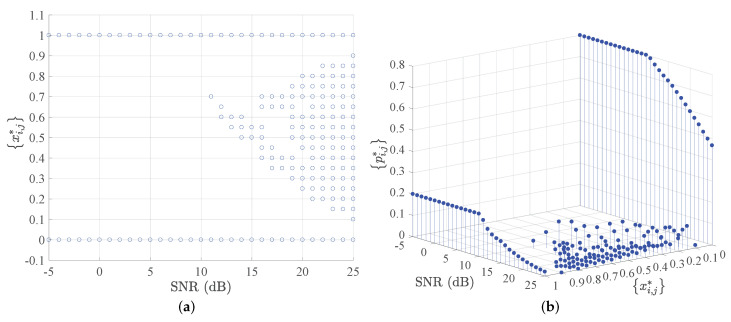
(**a**)The optimal input positions $\left\{x_{i,j}^{*}\right\}$ for different SNR with $\alpha =\frac{1}{5}$; (**b**)The optimal input distribution $\left\{x_{i,j}^{*},p_{i,j}^{*}\right\}$ for different SNR with $\alpha =\frac{1}{5}$.

We first consider the capacity region of VLC multiple access channels in the *general case*, i.e., both the peak optical power and average optical power constraints. As a simplification, we assume $\alpha_{1}=\alpha_{2}=\alpha ,\alpha \in \left(0,1\right)$ in the following simulations, which can also be selected independently. Figure 2a,b illustrate the optimal input positions $\left\{x_{i,j}^{*}\right\}$ and the optimal input discrete distribution $\left\{x_{i,j}^{*},p_{i,j}^{*}\right\}$ of VLC multiple access channels for different SNRs with $\alpha =\frac{1}{5}$, respectively. For the SNR ≤10 dB case, the optimal input positions have two discrete points, $\left\{0,A\right\}$, with unequal probability, i.e., $\left\{0.8,0.2\right\}$. Hence, the on-off keying (OOK) modulation can be applied as a practical method to achieve the capacity of VLC multiple access channels at low SNR. For SNR >10 dB, the optimal input positions also have more than two discrete points, which implies that the corresponding capacity-achieving modulator should be extended to the multiple plus amplitude modulation (PAM).

**Figure 3:**
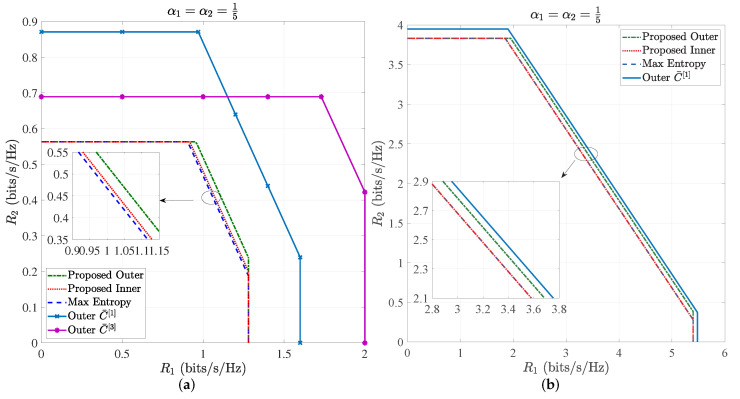
(**a**) Outer and inner bounds of capacity region of VLC multiple access channels with $\alpha =\frac{1}{5}$, $\frac{A_{1}}{\sigma }=10\;\mathrm{dB}$ and $\frac{A_{2}}{\sigma }=5\;\mathrm{dB}$; (**b**) outer and inner bounds of capacity region of VLC multiple access channels $\alpha =\frac{1}{5}$, $\frac{A_{1}}{\sigma }=25\;\mathrm{dB}$ and $\frac{A_{2}}{\sigma }=20\;\mathrm{dB}$.

Figure 3a,b illustrates the inner and outer bounds of the capacity region of VLC multiple access channels at low SNR, i.e., $\frac{A_{1}}{\sigma }=10\;\mathrm{dB}$ and $\frac{A_{2}}{\sigma }=5\;\mathrm{dB}$, and high SNR, i.e., $\frac{A_{1}}{\sigma }=25\;\mathrm{dB}$ and $\frac{A_{2}}{\sigma }=20\;\mathrm{dB}$, respectively, where $\alpha =\frac{1}{5}$. It can be observed from Figure 3a that the proposed outer bound is lower than existing outer bounds, i.e., ${\bar{C}}^{\left[1\right]}$ and ${\bar{C}}^{\left[3\right]}$, and the proposed inner bound is larger than the discrete entropy maximization inner bound (Max Entropy).

**Figure 4:**
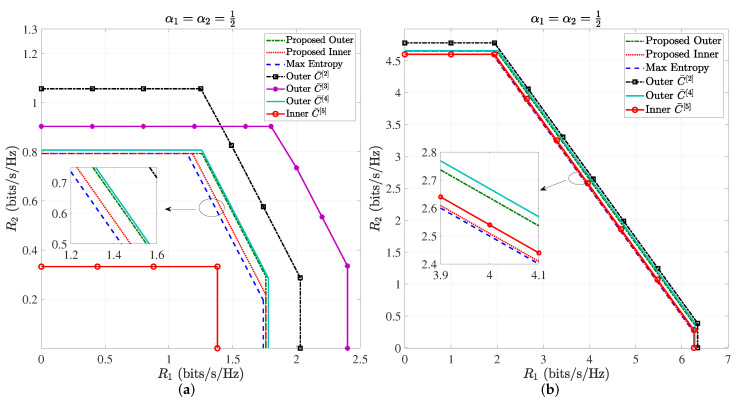
(**a**) Outer and inner bounds of capacity region of VLC multiple access channels with $\alpha =\frac{1}{2}$, $\frac{A_{1}}{\sigma }=10\;\mathrm{dB}$ and $\frac{A_{2}}{\sigma }=5\;\mathrm{dB}$; (**b**) outer and inner bounds of capacity region of VLC multiple access channels $\alpha =\frac{1}{2}$, $\frac{A_{1}}{\sigma }=25\;\mathrm{dB}$ and $\frac{A_{2}}{\sigma }=20\;\mathrm{dB}$.

Figure 4a,b shows the *special case*, i.e., only the peak optical power constraint, where $\alpha =\frac{1}{2}$. Both Figure 3 and Figure 4 demonstrate that the proposed outer bound is lowest among those outer bounds, while the proposed inner bound is higher than the exiting inner bounds except for the one side of the inner bound ${\bar{C}}^{\left[5\right]}$ at high SNR, i.e., the sum rate $R_{1}+R_{2}$ of ${\bar{C}}^{\left[5\right]}$ in Figure 4b.

**Figure 5:**
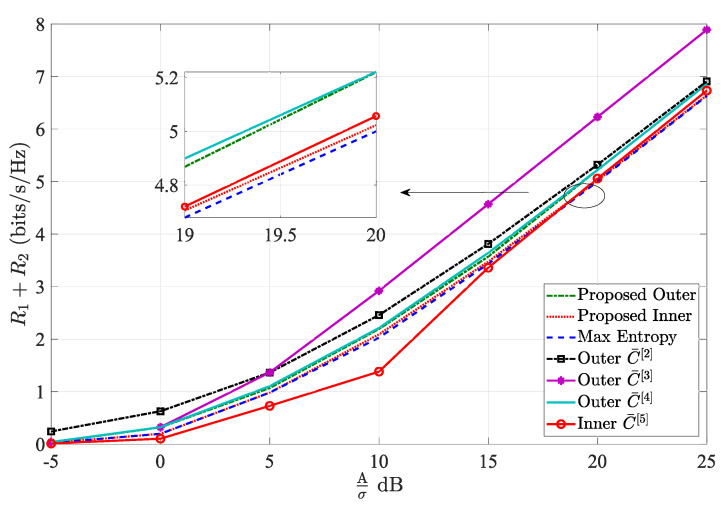
Sum rate of two users $R_{1}+R_{2}$ versus SNR (dB) with $\alpha =\frac{1}{2}$.

Figure 5 presents the sum rate of two users $R_{1}+R_{2}$ (bits/s/Hz) of proposed inner and outer bounds, discrete entropy maximization inner bound, ${\bar{C}}^{\left[2\right]}$, ${\bar{C}}^{\left[3\right]}$, ${\bar{C}}^{\left[4\right]}$ and ${\bar{C}}^{\left[5\right]}$ versus SNR (dB) at the special case, i.e., only the peak optical power constraint, where $\alpha =\frac{1}{2}$. We can observe that the proposed inner bound and the discrete entropy maximization inner bound are close at low SNR, while the proposed inner bound is higher than the discrete entropy maximization inner bound. This result verifies the conclusion that the entropy maximization distributions of $X_{1}$ and $X_{2}$ are not optimal input distributions, because $\max H\left(X_{1}\right)+\max H\left(X_{2}\right)$ cannot be guaranteed to maximize the differential entropy $h\left(X_{1}+X_{2}+Z\right)$. Meanwhile, it has been proven for the single-user channel that the achievable rate based on the discrete entropy maximization is always lower than the inexact gradient descent method [12]. Thus, the proposed inner bound can always achieve a higher sum rate than the discrete entropy maximization inner bound. Although ${\bar{C}}^{\left[5\right]}$ is highest at high SNR due to asymptotic optimality [10], the proposed inner bound is very closed to it. Meanwhile, ${\bar{C}}^{\left[5\right]}$ is worse at lower and moderate SNR, and the proposed inner bound is highest. If the two inner bounds can be jointed, a better inner bound can be obtained. However, ${\bar{C}}^{\left[5\right]}$ are established under either a per-user average-power constraint or a per-user peak-power constraint, and the performance is not good at lower SNR. Thus, the proposed inner bound can be more flexibly utilized for different constraints and SNR regions. Moreover, the proposed outer bound is lower than existing outer bounds ${\bar{C}}^{\left[2\right]}$, ${\bar{C}}^{\left[3\right]}$ and ${\bar{C}}^{\left[4\right]}$. Although ${\bar{C}}^{\left[4\right]}$ is closed to the proposed outer bound, it also only considers the peak-power constraint. Therefore, the performance of the proposed outer bound is better than others.

## 4. Conclusions

In this paper, we investigated the channel capacity region for the general VLC multiple access channel networks with both peak and average power constraints. Without loss of generality, we studied a two-user multiple access channel and provided a new inner bound and a new outer bound. According to the existing works for the single-user VLC channel, the capacity-achieving input signal should follow discrete distributions because of peak power constraints. Then, the exact capacity $\max I\left(X_{i};Y|X_{\bar{i}}\right)$ and optimal input distribution $P_{X_{i}}^{*}\left(x_{i}\right)$ are given by the inexact gradient descent method, $i=1,2,\bar{i}=3-i$. Furthermore, a new inner bound and a new outer bound for the sum rate $I\left(X_{1},X_{2};Y\right)$ are derived though the inexact gradient descent method. Finally, a new channel capacity region can be built from the combination of the above bounds. Numerical results show that the proposed outer bound is the tightest among existing benchmarks, and the proposed inner bound is the tightest in the lower and moderate SNR and very closed to the best bound in the high SNR. In theory, the proposed inner and outer bounds are more flexible to cover different constraints and SNRs. Besides, the proposed bounds can be easily extended to a case with more than two users. This paper developed a new inner bound and a new outer bound of the channel capacity region for the general VLC multiple access channel networks with both peak and average power constraints. However, the exact capacity of the sum rate is still unknown. In further works, we hope to find a tighter channel capacity region for the general VLC multiple access channel networks with both peak and average power constraints and apply the proposed bounds in the practical VLC networks.

## Data Availability

Not applicable.

## Abbreviations

The following abbreviations are used in this manuscript:

VLC	Visible light communication
SNR	Signal-to-noise ratio
RF	Radio frequency
LED	Light-emitting diodes
IM/DD	Intensity modulation and direct detection
BC	Broadcast channel
PD	Photon detector
PDF	Probability density function
OOK	On-off keying
PAM	Plus amplitude modulation
